# 胸部*SMARCA4*缺失性未分化肿瘤与*SMARCA4*缺失性非小细胞肺癌临床特征及预后分析

**DOI:** 10.3779/j.issn.1009-3419.2025.102.45

**Published:** 2025-12-20

**Authors:** Yingxue GUO, Jinlan YANG, Xiang LV, Xijun LIU, Fengxiang LI, Jinzhi WANG, Peng ZHANG, Jianbin LI, Wei WANG

**Affiliations:** ^1^257000 东营，胜利油田中心医院肿瘤科; ^1^Department of Oncology, Shengli Oilfield Central Hospital, Dongying 257000, China; ^2^250117 济南，山东第一医科大学附属肿瘤医院放疗科; ^2^Department of Radiation Oncology, Shandong First Medical University Affiliated Tumor Hospital, Jinan 250117, China; ^3^250117 济南，山东第一医科大学附属肿瘤医院病理科; ^3^Department of Pathology, Shandong First Medical University Affiliated Tumor Hospital, Jinan 250117, China

**Keywords:** 肺肿瘤, *SMARCA4*缺失, 临床特征, 免疫治疗, 放射治疗, 局部控制率, Lung neoplasms, *SMARCA4*-deficient, Clinical characteristics, Immunotherapy, Radiotherapy, Local control rate

## Abstract

**背景与目的:**

胸部*SMARCA4*缺失性未分化肿瘤（thoracic *SMARCA4*-deficient undifferentiated tumor, SMARCA4-UT）是2021年世界卫生组织（World Health Organization, WHO）第5版胸部肿瘤分类中新定义的一种上皮性肿瘤类型，发病率低，目前对其治疗及预后尚不明确。病理上可通过组织学形态、免疫组化与*SMARCA4*缺失性非小细胞肺癌（*SMARCA4*-deficient non-small cell lung cancer, SMARCA4-dNSCLC）相鉴别，但其临床特征、对放疗的敏感性以及预后是否有差异仍未可知。本研究通过分析SMARCA4-UT及SMARCA4-dNSCLC患者的临床特征，明确预后相关影响因素。

**方法:**

回顾性分析2022年6月至2025年2月山东第一医科大学附属肿瘤医院收治的SMARCA4-UT及SMARCA4-dNSCLC患者，患者均经病理确诊，且随访资料完整。统计分析两组患者临床病理特征及影像学表现的差异，并评估手术、放疗、免疫治疗及临床病理因素对两组患者预后的影响。

**结果:**

共27例SMARCA4-UT及40例SMARCA4-dNSCLC患者入组，两组患者在性别、吸烟史、年龄、肿瘤最大径、症状、分期、是否有胸膜转移及中性粒细胞/淋巴细胞计数比值（neutrophil to lymphocyte ratio, NLR）、系统免疫炎症指数（systemic immune-inflammation index, SII）方面均表现出相似的生物学特性。但两者好发部位有所差异，SMARCA4-UT更易发生在纵隔胸膜（22.22%）、右肺下叶（25.93%），而SMARCA4-dNSCLC更易发生在右肺上叶（25.00%）及左肺上叶（22.50%）（*P*<0.05）；SMARCA4-UT更常表现为局部浸润侵犯邻近结构且淋巴结转移更广泛（淋巴结转移≥5站者55.56% *vs* 27.50%）。两者对放疗的敏感性均高，放疗后6个月局部控制率（local control rate, LCR）分别为84.62% *vs* 83.33%，免疫治疗的客观缓解率（objective response rate, ORR）分别为91.67% *vs* 68.18%，均无统计学差异（*P*>0.05）。生存方面，两组患者在无进展生存期（progression-free survival, PFS）与总生存期（overall survival, OS）方面均未表现出显著差异。*Cox*多因素回归分析显示，手术可以改善这两类患者的预后，而NLR≥3.57为其预后不良预测因子。

**结论:**

SMARCA4-UT与SMARCA4-dNSCLC具有相似的临床特征，生存预后相似，在局部浸润侵犯邻近结构、纵隔淋巴结转移及肿瘤原发部位上略有不同。手术可以改善两组患者的生存，且两者对放疗均表现出高的敏感性，但放疗未表现出患者的生存获益。治疗前NLR值可作为预后预测指标。

## Abstract (en)

**Background and objective:**

Thoracic *SMARCA4*-deficient undifferentiated tumor (SMARCA4-UT) is a newly defined type of epithelial tumor in the 2021 World Health Organization (WHO) fifth edition classification of thoracic tumors, with a low incidence. Currently, its treatment and prognosis remain unclear. Pathologically, it can be distinguished from *SMARCA4*-deficient non-small cell lung cancer (SMARCA4-dNSCLC) based on histological morphology and immunohistochemistry, yet whether there are differences in their clinical features, sensitivity to radiotherapy, and prognosis remains unknown. This study aimed to analyze the clinical characteristics of patients with SMARCA4-UT and SMARCA4-dNSCLC and to identify prognostic factors.

**Methods:**

A retrospective analysis was performed on pathologically confirmed SMARCA4-UT and SMARCA4-dNSCLC patients with complete follow-up data who were admitted to Shandong First Medical University Affiliated Tumor Hospital from June 2022 to February 2025. The differences in clinicopathological characteristics and imaging findings between the two groups were statistically analyzed, and the impacts of surgery, radiotherapy, immunotherapy, and clinicopathological factors on the prognosis of patients in both groups were assessed.

**Results:**

A total of 27 SMARCA4-UT patients and 40 SMARCA4-dNSCLC patients were enrolled. Both groups showed similar biological characteristics in terms of gender, age, smoking history, tumor size, symptoms, stage, presence of pleural metastasis, neutrophil-to-lymphocyte ratio (NLR), and systemic immune-inflammation index (SII). However, there were differences in the predilection sites: SMARCA4-UT occurred more frequently in the mediastinal pleura (22.22%) and right lower lobe (25.93%), while SMARCA4-dNSCLC occurred more frequently in the right upper lobe (25.00%) and left upper lobe (22.50%) (*P*<0.05). Furthermore, SMARCA4-UT more often presented with local invasion into adjacent structures and more extensive lymph node metastasis (proportion with metastasis in ≥5 lymph node stations: 55.56% *vs* 27.50%). Both types showed high sensitivity to radiotherapy, with a 6-month local control rate (LCR) of 84.62% *vs* 83.33% after radiotherapy; the objective response rate (ORR) of immunotherapy was 91.67% *vs* 68.18% (*P*>0.05). Regarding survival, the two groups did not show significant differences in progression-free survival (PFS) or overall survival (OS). *Cox* multivariate regression analysis indicated that surgery could improve the prognosis of both types, while a high NLR (≥3.57) was a predictor of poor prognosis.

**Conclusion:**

SMARCA4-UT and SMARCA4-dNSCLC share similar clinical characteristics and survival outcomes, with minor differences in local invasion, mediastinal lymph node metastasis, and primary tumor location. Surgery improved survival in both groups. Although both tumor types exhibited high sensitivity to radiotherapy, this did not translate into a significant survival benefit for the patients. Pretreatment NLR is a potential prognostic indicator.

在*SMARCA4*缺失的状态下，细支气管外分泌细胞对恶性转化和肿瘤进展敏感，导致高度恶性的去分化肿瘤和转移发生率增加^[1]^。非小细胞肺癌（non-small cell lung cancer, NSCLC）中5%-10%的患者会携带*SMARCA4*缺失突变，命名为*SMARCA4*缺失性NSCLC（*SMARCA4*-deficient NSCLC, SMARCA4-dNSCLC）^[2,3]^。胸部*SMARCA4*缺失性未分化肿瘤（*SMARCA4*‐deficient undifferentiated tumors, SMARCA4-UT）是在2021年世界卫生组织（World Health Organization, WHO）第5版胸部肿瘤分类中新定义的一种独立的疾病实体，其核心特征正是“未分化”，并表现出*SMARCA4*（*BRG1*）缺陷。两组患者均具有恶性度高、侵袭性强、预后差的特点^[4]^，SMARCA4-UT由于其独特的组织学、免疫组化、临床和预后特征，作为不同于SMARCA4-dNSCLC的独特实体被区分。但因SMARCA4-UT较为罕见，针对两者的临床特征、免疫治疗及放疗有效性对比研究较少，且未有针对放疗敏感性及预后预测因子的相关研究。因此，本研究回顾性分析了SMARCA4-UT及SMARCA4-dNSCLC患者的临床病理、影像学特征和局部控制及生存情况，旨在评估不同病理类型下不同治疗模式的疗效差异以及对预后的影响，为两类肿瘤的临床管理提供依据。

## 1 资料与方法

### 1.1 病例选择

收集2022年6月至2025年2月山东第一医科大学附属肿瘤医院收治的胸部SMARCA4-UT及SMARCA4-dNSCLC患者。根据WHO第5版胸部肿瘤分类标准，所有病理诊断均经2名5年以上病理医师审核确认为*SMARCA4*（*BRG1*）表达阴性或部分表达阴性的肺部肿瘤患者，剔除诊断不明确或者存在明显异质性的病例，以确保后续研究的准确性和可重复性，按照国际抗癌联盟第8版肺癌肿瘤原发灶-淋巴结-转移（tumor-node-metastasis, TNM）分期标准进行分期。排除标准：（1）非初诊患者，或初诊治疗前无完善的影像学检查；（2）临床资料不全或随访时间不足：随访时间需≥6个月。最终符合入组条件患者有67例，其中27例胸部SMARCA4-UT及40例SMARCA4-dNSCLC患者。

本研究为回顾性研究，获山东第一医科大学附属肿瘤医院伦理委员会批准（批准号：SDTHEC 202508040），患者知情同意豁免。

### 1.2 临床病理资料收集

（1）患者的一般信息：性别、年龄、吸烟史（吸烟指数）、发病症状；（2）实验室检查：淋巴细胞计数、血小板计数（platelet, PLT）、中性粒细胞计数、中性粒细胞-淋巴细胞比值（neutrophil-to-lymphocyte ratio, NLR）、全身免疫炎症指数（systemic immune-inflammation index, SII）（计算公式：SII=PLT×NLR）及肿瘤标志物[癌胚抗原（carcinoembryonic antigen, CEA）、细胞角蛋白19片段（cytokeratin 19 fragment, CYFRA21-1）、神经元特异性烯醇化酶（neuron-specific enolase, NSE）、胃泌素释放肽前体（pro-gastrin-releasing peptide, ProGRP）、鳞状上皮细胞癌抗原（squamous cell carcinoma antigen, SCC）]；（3）影像检查：通过初次诊断强化计算机断层扫描（computed tomography, CT）、颅脑磁共振成像（magnetic resonance imaging, MRI）、骨放射性核素骨扫描（emission computed tomography, ECT）或正电子发射型计算机断层显像（positron emission tomography/CT, PET/CT）表现，明确肿瘤大小、发病部位与周围组织器官的关系，确定肿瘤临床分期；（4）明确Ki-67水平、程序性细胞死亡配体1（programmed cell death ligand 1, PD-L1）表达情况、下一代测序（next-generation sequencing, NGS）结果。并详细记录治疗方案。

### 1.3 随访

通过门诊或电话随访统计患者生存状况。总生存期（overall survival, OS）定义为从疾病首次确诊至因任何原因引起死亡或最后一次随访的时间间隔，无进展生存期（progression-free survival, PFS）定义为从初次诊断到肿瘤复发进展和死亡的时间间隔，随访终止日期仍存活或失访定义为删失。本研究随访截止日期为2025年9月27日，随访时间为0.7-39.9个月，中位随访时间为9个月。。

### 1.4 统计学方法

采用R 4.3.2软件进行数据分析。组间计量数资料比较采用独立样本*t*检验（符合正态分布）及*Mann-Whitney U*检验（非正态分布资料）。计数资料组间比较采用卡方检验或*Fisher*确切概率法。生存分析采用*Kaplan-Meier*法，组间比较采用*Log-rank*检验。预后影响因素分析采用*Cox*比例风险回归模型，先进行单因素分析筛选变量（*P*<0.10），经共线性诊断后纳入多因素模型。所有检验均为双侧，*P*<0.05为差异有统计学意义。

## 2 结果

### 2.1 两组一般临床资料比较

两组患者一般情况及影像学表现见表1，典型病理图示见图1。基于对胸部SMARCA4-UT（*n*=27）和SMARCA4-dNSCLC（*n*=40）患者基线特征的比较分析：两组在性别、年龄、吸烟史、初诊症状、临床分期、远处转移及胸膜转移方面均无显著差异（*P*>0.05），均以中老年男性、重度吸烟者为主，常表现为肺部症状，诊断时多为晚期，且常见胸膜转移及远处转移。然而，在发病部位、局部侵犯程度及淋巴结转移范围方面存在显著差异（*P*<0.05）：SMARCA4-UT更多见于纵隔胸膜及右肺下叶，更常广泛侵犯邻近结构（如食管、支气管），且出现≥5站淋巴结转移的比例显著更高（55.56% *vs* 27.50%, *P*=0.040）。SMARCA4-dNSCLC则更常见于右肺上叶及左肺上叶。此外，两组患者分别有20及24例患者进行了Ki-67的检测，发现Ki-67高表达（≥50%）在SMARCA4-UT组更为常见[90.00% (18/20) *vs* 70.83% (17/24)]。

**表 1:** 胸部SMARCA4-UT组及SMARCA4-dNSCLC组患者临床特征对比

Index	Total	SMARCA4-UT (*n*=27)	SMARCA4-dNSCLC (*n*=40)	*P*
Age (yr)	64.38±8.11	64.35±8.02	67.42±8.03	0.129
Gender				0.679
Female	6 (8.96%)	3 (11.11%)	3 (7.50%)	
Male	61 (91.04%)	24 (88.89%)	37 (92.50%)	
Smoking				0.577
No smoking	18 (26.87%)	9 (33.33%)	9 (22.50%)	
Light smoking (<30 pack-years)	8 (11.94%)	3 (11.11%)	5 (12.50%)	
Heavy smoking (≥30 pack-years)	41 (61.19%)	15 (55.56%)	26 (65.00%)	
Maximum tumor diameter (cm)	4.10 (2.70, 6.70)	3.70 (2.55, 6.65)	4.55 (2.90, 6.75)	0.518
Initial symptoms				0.757
Asymptomatic	14 (20.90%)	5 (18.52%)	9 (22.50%)	
Pulmonary symptoms	36 (53.73%)	16 (59.26%)	20 (50.00%)	
Systemic and metastatic symptoms	17 (25.37%)	6 (22.22%)	11 (27.50%)	
Location of lesion				0.033
Right upper lobe	14 (20.90%)	4 (14.81%)	10 (25.00%)	
Right middle lobe	4 (5.97%)	3 (11.11%)	1 (2.50%)	
Right lower lobe	13 (19.40%)	7 (25.93%)	6 (15.00%)	
Left upper lobe	9 (13.43%)	0 (0.00%)	9 (22.50%)	
Left lower lobe	11 (16.42%)	5 (18.52%)	6 (15.00%)	
Hilar/central type	7 (10.45%)	2 (7.41%)	5 (12.50%)	
Mediastinum/pleura	9 (13.43%)	6 (22.22%)	3 (7.50%)	
TNM				0.999
I-IIIA	6 (8.96%)	2 (7.41%)	4 (10.00%)	
IIIB-IIIC	16 (23.88%)	7 (25.92%)	9 (22.50%)	
IV	45 (67.16%)	18 (66.67%)	27 (67.50%)	
Invasion of adjacent organs				0.037
Absent	50 (74.63%)	16 (59.26%)	34 (85.00%)	
Present	17 (25.37%)	11 (40.74%)	6 (15.00%)	
Lymph node stations involved				0.040
<5 stations	41 (61.19%)	12 (44.44%)	29 (72.50%)	
≥5 stations	26 (38.81%)	15 (55.56%)	11 (27.50%)	
Distant metastasis				0.163
Absent	34 (50.75%)	17 (62.96%)	17 (42.50%)	
Present	33 (49.25%)	10 (37.04%)	23 (57.50%)	
Pleural metastasis				0.786
Absent	52 (77.61%)	20 (74.07%)	32 (80.00%)	
Present	15 (22.39%)	7 (25.93%)	8 (20.00%)	
NLR				0.913
<3.57	39 (58.21%)	15 (55.56%)	24 (60.00%)	
≥3.57	28 (41.79%)	12 (44.44%)	16 (40.00%)	
SII				0.999
<660	21 (31.34%)	8 (29.63%)	13 (32.50%)	
≥660	46 (68.66%)	19 (70.37%)	27 (67.50%)	

SMARCA4-UT: thoracic *SMARCA4*-deficient undifferentiated tumor; SMARCA4-dNSCLC: *SMARCA4*-deficient non-small cell lung cancer; TNM: tumor-node-metastasis; NLR: neutrophil-to-lymphocyte ratio; SII: systemic immune-inflammation index.

**图 1:**
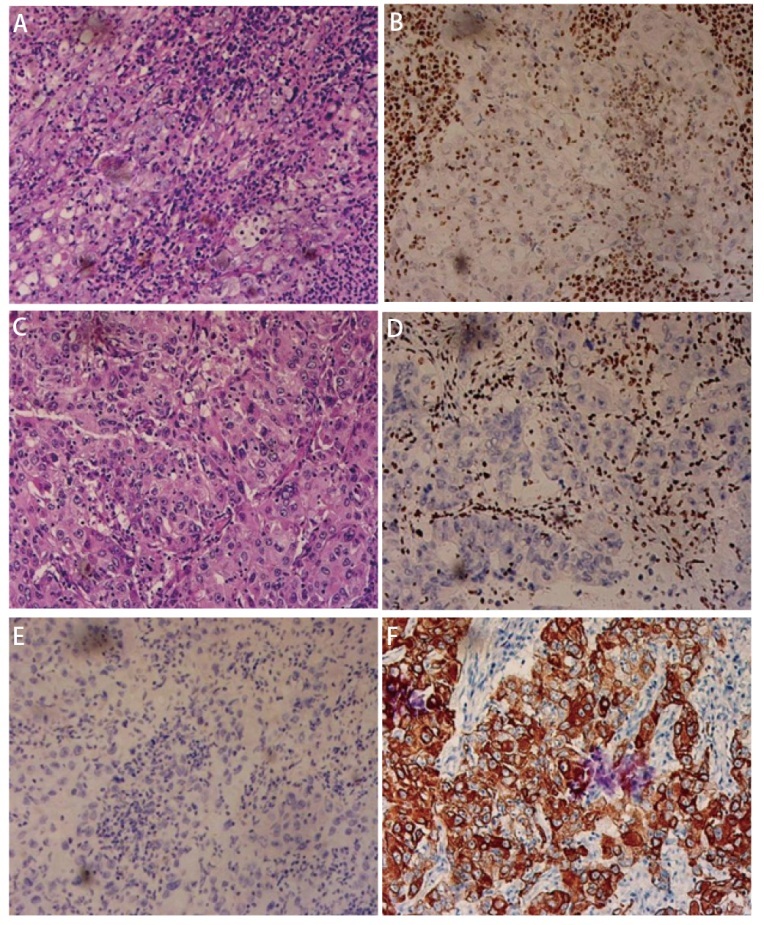
SMARCA4-UT及SMARCA4-dNSCLC（分化差腺癌）典型病理图片。A：SMARCA4-UT HE染色（×20）；B：SMARCA4-UT免疫组化染色（SMARCA4, ×20）显示肿瘤细胞核表达缺失；C：SMARCA4-dNSCLC（分化差腺癌）HE染色（×20）；D：SMARCA4-dNSCLC（分化差腺癌）免疫组化染色（SMARCA4, ×20）显示肿瘤细胞核表达缺失；E：SMARCA4-dNSCLC（分化差腺癌）免疫组化染色（P40, ×20）显示肿瘤细胞核表达阴性；F：SMARCA4-dNSCLC（分化差腺癌）免疫组化染色（CK7, ×20）显示肿瘤细胞胞质阳性表达。

### 2.2 肿瘤标志物

胸部SMARCA4-UT组：25例检测，无异常者6例，NSE升高者19例，CYFRA21-1升高者8例，CEA升高者3例，ProGRP升高者2例，SCC有1例异常升高。SMARCA4-dNSCLC组：39例检测，无异常者3例，CEA升高者28例，CYFRA21-1升高者25例，并与CEA升高呈高度一致性；NSE、ProGRP、SCC升高者分别有18、2、4例。SMARCA4-UT组单一NSE升高患者多，而SMARCA4-dNSCLC组多合并多种肿瘤标志物升高，可为其病理前诊断提供参考。

### 2.3 NGS结果

胸部SMARCA4-UT组中12例患者完善NGS，6例患者无基因突变，1例（16.67%）患者*SMARCA4*突变，合并人表皮生长因子受体-2（human epidermal growth factor receptor 2, *HER-2*）突变、表皮生长因子受体（epidermal growth factor receptor, *EGFR*）外显子21 L858R突变、间质上皮细胞转化因子（mesenchymal-epithelial transition factor, *MET*）融合突变均1例（8.33%），2例（16.67%）患者*TP53*突变，1例（8.33%）患者丝氨酸/苏氨酸激酶11（serine/threonine kinase 11, *STK11*）突变，1例（8.33%）患者磷脂酰肌醇-3-激酶催化亚单位α（phosphoinositide-3-kinase catalytic alpha polypeptide, *PIK3CA*）突变；8例患者完善PD-L1检测，阴性表达3例（37.50%），低表达（1%-49%）5例（62.50%），无高表达（≥50%）患者。

SMARCA4-dNSCLC组中31例患者完善NGS，17例患者无基因突变，3例（9.68%）患者发现*SMARCA4*突变，6例（19.35%）患者合并Kirsten大鼠肉瘤病毒癌基因同源物（Kirsten rat sarcoma viral oncogene homolog, *KRAS*）突变，2例（6.45%）患者*EGFR*突变，1例（3.23%）患者*BRAF*融合突变，7例（22.58%）患者*TP53*突变，1例（3.23%）患者*STK11*突变，2例（6.45%）患者*PIK3CA*突变；33例患者完善PD-L1检测，阴性表达8例（24.24%），低表达（1%-49%）22例（66.67%），高表达（≥50%）3例（9.09%）。SMARCA4-dNSCLC组与SMARCA4-UT组比较无统计学差异（*P*>0.05）。

### 2.4 治疗及预后

#### 2.4.1 放疗

胸部SMARCA4-UT组接受放疗患者共12例，其中1例患者行原发灶及肾上腺两程放疗。原发灶放疗9例，其中6例部分缓解（partial response, PR），2例疾病稳定（stable disease, SD），1例疾病进展（progressive disease, PD）。2例行骨转移灶放疗，疼痛明显减轻，疗效评价PR；脑转移灶放疗1例，疗效评估PR；肾上腺放疗1例，疗效评估为完全缓解（complete response, CR）。放疗后1个月局部控制率（local control rate, LCR）为92.31%（12/13），6个月LCR为84.62%（11/13）（有1例患者不同时间经历了2个部位的放疗，计算LCR时总病变数量为13）。SMARCA4-dNSCLC组放疗患者共20例，原发灶放疗8例，5例PR，2例SD，1例PD；7例骨转移灶放疗，1例PD，1例PR，5例SD；3例脑转移放疗，1例SD，2例PR；1例右锁骨上、纵隔2R区转移淋巴结放疗，达PR；1例肝脏、肾上腺转移灶放疗，达SD。放疗后1个月LCR为90.00%（18/20），6个月LCR为83.33%（10/12），余为死亡、影像失访不可评效。两组患者放疗对LCR无统计学差异（*P*>0.05）。图2为SMARCA4-UT及SMARCA4-dNSCLC典型患者放疗前后对比图像。

**图 2:**
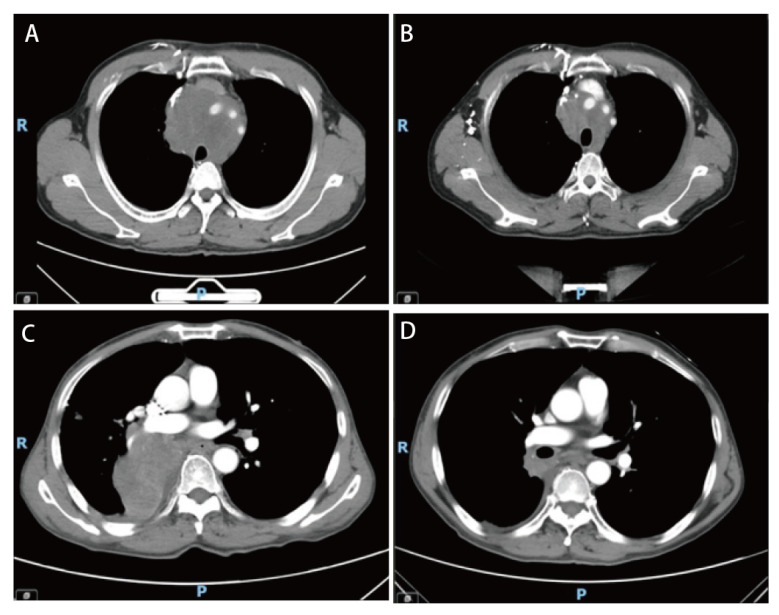
SMARCA4-UT与SMARCA4-dNSCLC患者放疗前后CT影像对比。A、B：SMARCA4-UT患者放疗前（A）与放疗后（B）的轴位CT图像；C、D：SMARCA4-NSCLC患者放疗前（C）与放疗后（D）的轴位CT图像。

#### 2.4.2 免疫治疗

胸部SMARCA4-UT组接受免疫治疗患者共17例（62.96%），9例PR，2例SD，1例PD，5例未评效，客观缓解率（objective response rate, ORR）为91.67%。SMARCA4-dNSCLC组接受免疫治疗患者共26例（65.00%），13例PR，2例SD，7例PD，4例未评效，ORR为68.18%。两组免疫治疗反应上差异无统计学意义（*P*=0.21）。

#### 2.4.3 生存分析

为比较胸部SMARCA4-UT与SMARCA4-dNSCLC患者的生存预后，本研究采用*Kaplan-Meier*法绘制两组的PFS曲线与OS曲线，并计算中位生存时间。组间生存差异通过*Log-rank*检验进行评估。结果显示（图3），胸部SMARCA4-UT组与SMARCA4-dNSCLC组的中位PFS分别为6.0与9.0个月，中位OS分别为13.0与17.0个月。然而，*Log-rank*检验显示两组间在PFS与OS上的差异无统计学意义（*P*值均为0.990）。应用*Cox*回归模型单因素分析筛选患者的预后影响因素，因病例数较少，选取*P*<0.1的因素包括：NLR（≥3.57）、SII（≥660）、一线应用免疫抑制剂、IV期、手术、性别。将其纳入*Cox*回归模型进行多因素分析（表2），结果显示NLR（≥3.57）可使胸部SMARCA4-UT及SMARCA4-dNSCLC患者死亡风险升高（RR=2.793）。手术治疗为其改善预后治疗方式，可降低死亡风险（RR=0.120）。

**图 3:**
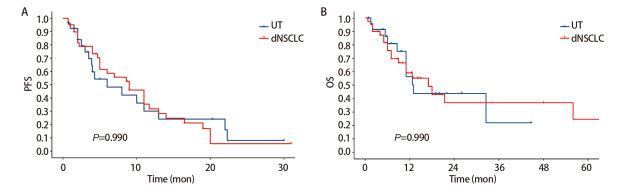
SMARCA4-UT及SMARCA4-dNSCLC生存曲线。A：PFS；B：OS。

**表 2:** SMARCA4-UT及SMARCA4-dNSCLC预后影响因素的回归模型

Prognostic factors	RR	95%CI	*P*
Stage IV (Yes *vs* No)	1.191	0.451-3.148	0.724
Surgery (Yes *vs* No)	0.120	0.016-0.919	0.041
NRL≥3.57 (Yes *vs* No)	2.793	1.016-7.674	0.046
SII≥660 (Yes *vs* No)	1.095	0.330-3.633	0.882
Immunotherapy (Yes *vs* No)	0.487	0.231-1.027	0.059
Gender (Male *vs* Female)	4.779	0.932-24.497	0.061

RR: relative risk.

## 3 讨论

SMARCA4-UT曾归类为“*SMARCA4*缺失性胸部肉瘤（*SMARCA4*-deficient thoracic sarcoma, SMARCA4-DTS）”，后证实这一类肿瘤的组织起源是肺组织^[5]^，但其转录组特征与肺癌显著不同，而与卵巢高钙血症型小细胞癌（small-cell carcinoma of the ovary, hypercalcemic type-genetics, SCCOHT）及恶性横纹肌样瘤（malignant rhabdoid tumor, MRT）相似，因此将其重新命名，归为“其他肺上皮肿瘤”类型。值得注意的是，部分NSCLC患者也会出现*SMARCA4*基因缺失^[2,3]^。然而，两者在组织起源、细胞形态、免疫表型及分子特征等方面存在根本性差异。但因发病率低且缺乏对比研究，其临床异质性尚未明晰。本研究首次系统比较了两类肿瘤的临床特征、治疗反应及预后因素。

既往研究中，胸部SMARCA4-UT及SMARCA4-dNSCLC均常发生在中年男性，并与吸烟史密切相关^[6,7]^。但本研究胸部SMARCA4-UT及SMARCA4-dNSCLC患者更好发于老年，这与吴方君等^[8]^分析结果具有相似性，发病年龄的差异性不除外人种差异所导致。胸部SMARCA4-UT及SMARCA4-dNSCLC首发症状均多为咳嗽、咳痰、喘憋、痰中带血等肺部症状，发病时多为晚期，合并远处转移、部分有胸膜转移及局部侵犯、压迫症状，与文献报道^[6,8]^相符。胚胎起源或局部微环境的差异可能导致不同疾病发病部位的差异，胸部SMARCA4-UT可能起源于具有多向分化潜能的原始细胞^[9]^，发病部位更倾向于累及纵隔胸膜及右肺下叶，而好发于双肺上叶的SMARCA4-dNSCLC更倾向起源于经典支气管上皮^[10]^。高表达Ki-67的NSCLC增殖更快、侵袭性更强，与死亡和复发风险增加关联^[11]^，胸部SMARCA4-UT及SMARCA4-dNSCLC两组患者中Ki-67阳性率>50%者高达90.00%和70.83%，这种处于活跃分裂状态的肿瘤细胞，更易发生侵袭和转移，这也解释了其在初诊时更易合并远处转移和更广泛的淋巴结转移的特性。

放疗通过诱导DNA损伤而提高LCR，而*SMARCA4*作为SWI/SNF复合体的核心亚基，其缺失可能导致染色质重塑异常，使肿瘤细胞修复放疗损伤的能力下降。本研究发现，两组患者对放疗均具有较高的敏感性，放疗后6个月的LCR均超过83%。但放疗并未显著改善两组患者的生存期，这与既往研究^[12,13]^一致。降低肿瘤负荷、提高ORR和延缓肿瘤进展在抗肿瘤治疗中具有重要的临床意义，但由于*SMARCA4*缺失更多合并远处转移，放疗以减轻症状为主要目的，放疗的PFS获益需叠加有效的全身治疗效果才能体现OS获益。基因组分析显示，胸部SMARCA4-UT与SMARCA4-dNSCLC在突变谱上存在显著重叠，常见突变包括*TP53*、*STK11*、Kelch样ECH相关蛋白1（Kelch-like ECH-associated protein 1, *KEAP1*）及*KRAS*等，这些共存突变可能进一步影响肿瘤的生物学行为及治疗反应^[14]^。然而，SMARCA4-UT的独特性在于常伴随*SMARCA2*的共缺失以及干细胞标志物（如*SOX2*、*CD34*、*SALL4*）的高频表达^[5]^。*SMARCA4*缺失与高肿瘤突变负荷（tumor mutational burden, TMB）和其他基因突变（如*TP53*、*KRAS*等）相关，这些特征可能导致肿瘤对传统治疗方法的耐药性增加^[15]^。因此，针对高度恶性的*SMARCA4*缺失肿瘤，尽管放疗可能在短期内缓解症状并控制局部病灶，但其对长期生存的影响有限。因此，未来研究应集中于探索更为有效的治疗策略，如放疗联合免疫治疗或靶向治疗，以改善*SMARCA4*缺失肿瘤患者的预后^[16]^。

*SMARCA4*缺失突变的胸部肿瘤患者，免疫检查点抑制剂（immune checkpoint inhibitors, ICIs）对患者治疗的生存优势目前研究结论并不统一^[3,17,18]^，需更多的研究证实或筛选免疫获益的亚组人群。*TP53*突变可能会导致TMB高表达，使用ICIs疗效更好^[17]^。与*KRAS*共突变相关的*SMARCA4*缺失比单独的*SMARCA4*突变预后更差，而SMARCA4-dNSCLC相较于胸部SMARCA4-UT，其*KRAS*突变率较高。*SMARCA4*与*KRAS*共突变时NSCLC组织中程序性细胞死亡受体1（programmed cell death protein 1, PD-1）^+^CD8^+^ T细胞数量减少^[18]^，而较高水平PD-1^+^CD8^+^ T细胞有助于提高ICIs的疗效，这可能是SMARCA4-dNSCLC中SMARCA4与*KRAS*共突变患者预后不佳的原因，而胸部SMARCA4-UT的ICIs治疗相对更有优势性。一线使用ICIs患者治疗有效率虽高，却并未改善患者生存，提示其更易耐药致病情进展。Wang等^[19]^认为*SMARCA4*缺失会导致肿瘤微环境中树突状细胞和CD4 T细胞的浸润显著减少，包括干扰素基因刺激因子（stimulator of interferon genes, STING）和白细胞介素-1β（interleukin-1β, IL-1β）在内的内源性免疫系统关键组成部分及关键炎症因子的表达下调，从而导致ICIs的效果减弱。然而，本研究仅少数患者接受免疫治疗及放疗，未来需进一步探索放疗联合ICIs的协同效应。

血液中的中性粒细胞一方面可以释放细胞因子和趋化因子，改变循环肿瘤细胞转移的微环境^[20]^，另一方面其可能直接与循环肿瘤细胞相互作用，导致淋巴细胞数量下降，进而加速肿瘤生长、转移^[21]^。PLT则可以通过激活转化生长因子-β1（transforming growth factor-β1, TGF-β1）/Smad和核因子-κB（nuclear factor-kappaB, NF-κB）信号通路，促使肿瘤细胞发生上皮-间质转化，增强其与其他细胞的黏附能力^[22]^。因此，NLR和SII反映全身炎症及免疫状态，与肿瘤发生、转移的多个环节密切关联。Tong等^[23]^在晚期NSCLC患者中发现高SII与铂类化疗耐药性有关，SII≥660、NLR≥3.57可以预测晚期NSCLC患者的不良预后。本研究中基于文献报道截断值进行生存分析，单因素分析显示两者均对生存呈负性影响；多因素分析显示，NLR（≥3.57）可使SMARCA4-UT及SMARCA4-dNSCLC患者死亡风险升高179.3%（RR=2.793），提示NLR作为一种潜在的生物标志物，在预测*SMARCA4*缺失患者的预后中具有重要作用，可以为患者提供更为精准的管理方案的预测。

未来的研究应继续探索手术与其他治疗的最佳组合，以进一步提高*SMARCA4*缺失突变的胸部肿瘤患者的生存率和生活质量。但本研究为单中心回顾性研究存在一定局限性，由于病例数量有限，且入组患者91%为穿刺病理活检证实，肿瘤固有的空间异质性体现不完善，在治疗压力下驱动的克隆演化导致时间异质性不可避免，因此，病理类型会存在一定的转化。在临床诊疗中，尤其在治疗前后或疾病进展时，多部位、深层次的病理活检，对于全面揭示此类肿瘤的异质性本质、避免诊断偏差具有重要意义。另一方面，合并驱动基因突变的患者接受靶向治疗，可能会干扰生存分析。再者，本研究主要依据免疫组化结果判定*SMARCA4*缺失状态，未能对全部病例进行*SMARCA4*基因测序，因此在精确区分I型与II型突变上存在局限。未来需要进行更大规模的多中心研究、更深入的基因型分析来验证我们的结论，并进一步探索两种肿瘤的个体化临床管理策略。
